# Polymeric nanocarriers delivery systems in ischemic stroke for targeted therapeutic strategies

**DOI:** 10.1186/s12951-024-02673-4

**Published:** 2024-07-18

**Authors:** Lin Zhu, Weijie Zhong, Xuchen Meng, Xiaosheng Yang, Wenchuan Zhang, Yayuan Tian, Yi Li

**Affiliations:** Department of Neurosurgery, Ninth People Hospital, Affiliated to Shanghai Jiao Tong University School of Medicine, Shanghai, 200011 People’s Republic of China

**Keywords:** Nanomedicine, Polymeric nanocarrier, Drug delivery system, Ischemic stroke, Targeted therapy

## Abstract

Ischemic stroke is a complex, high-mortality disease with multifactorial etiology and pathogenesis. Currently, drug therapy is mainly used treat ischemic stroke in clinic, but there are still some limitations, such as limited blood-brain barrier (BBB) penetration efficiency, a narrow treatment time window and drug side effects. Recent studies have pointed out that drug delivery systems based on polymeric nanocarriers can effectively improve the insufficient treatment for ischemic stroke. They can provide neuronal protection by extending the plasma half-life of drugs, enhancing the drug’s permeability to penetrate the BBB, and targeting specific structures and cells. In this review, we classified polymeric nanocarriers used for delivering ischemic stroke drugs and introduced their preparation methods. We also evaluated the feasibility and effectiveness and discussed the existing limitations and prospects of polymeric nanocarriers for ischemic stroke treatment. We hoped that this review could provide a theoretical basis for the future development of nanomedicine delivery systems for the treatment of ischemic stroke.

**Graphical Abstract:**

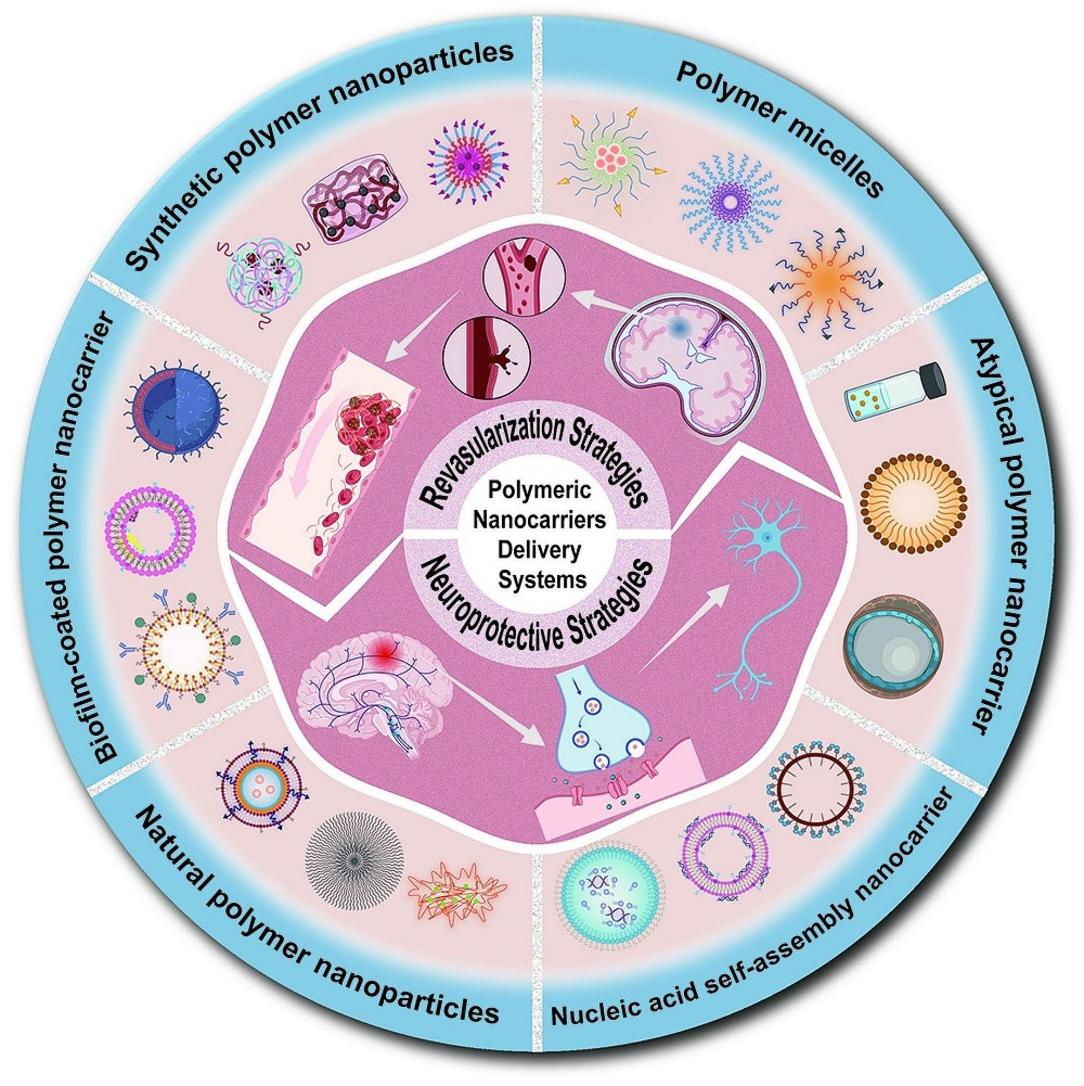

## Introduction

Ischemic stroke is the second leading cause of death globally, directly leading to 5.9 million deaths and indirectly resulting in 102 million disability adjusted deaths each year [1, 2]. It primarily results from the obstruction of cerebral blood vessels, leading to a reduction or interruption in the blood supply to brain tissue [3–7]. This insufficiency in blood supply diminishes the delivery of essential nutrients and oxygen, thereby inducing a hypoxic state in the brain tissue. Then, the hypoxic condition promotes the recruitment of leukocytes to the affected areas, exacerbating oxidative stress and inflammatory damage, further compounding cerebral tissue injury. In addition, damaged neurons and astrocytes generate reactive oxygen species (ROS), which in turn aggravate damage to neuronal cells and blood vessels. As a result, Prolonged oxidative stress and inflammatory responses ultimately may lead to the disruption of the blood-brain barrier (BBB), further deteriorating brain tissue and potentially culminating in cerebral parenchymal necrosis [8–10].

Recombinant tissue plasminogen activator (rtPA) presently stands as the exclusive therapeutic agent for ischemic stroke endorsed by the Food and Drug Administration [11]. Nevertheless, this conventional pharmacological modality is not devoid of limitations. Primarily, the stringent 4.5-hour therapeutic window for administering rtPA significantly restricts the temporal scope for effective stroke intervention. Furthermore, the intricate and dense architecture of the BBB impedes the transference of numerous thrombolytic, antioxidant, and neuroprotective pharmacological agents [12]. Additionally, the challenges encompassing the brief biological half-life of these agents, inadequate targeting following cerebral administration, and the potential secondary reperfusion injuries consequent to the reinstatement of blood flow in ischemic cerebral regions are yet to be surmounted [13]. Therefore, forging an efficacious and precision-targeted drug delivery system has emerged as an imperative research trajectory in the realm of ischemic stroke therapeutics.

In light of the relentless progress in medical nanomaterial sciences and their burgeoning integration into contemporary medical applications, it has become feasible to devise and implement a sophisticated nanoparticle-based drug delivery platform specifically tailored for intracerebral therapeutic interventions [14, 15]. Nanoparticles are methodically classified into three fundamental categories based on their chemical composition: organic, inorganic, and lipid-based nanoparticles. Within the organic subset, polymeric nanoparticles are distinguished by their optimal particle size and customizable surface properties, enabling efficient penetration through the BBB. Furthermore, these polymeric nanoparticulate systems can undergo targeted modifications to enhance their aggregation at specific therapeutic sites, thereby minimizing the nonspecific distribution of pharmacological agents [16, 17]. In the context of ischemic stroke, polymer nanoparticles have emerged as pivotal carriers in novel drug therapy delivery systems. The core therapeutic approaches facilitated by these ischemic stroke nanodrug delivery systems encompass revascularization and neuroprotection strategies, marking a significant leap in targeted treatment modalities [18–20].

In this review, we introduced the latest designs in polymeric nanocarrier delivery systems. These systems were developed based on a profound understanding of the pathological mechanisms, basic strategies, and limitations inherent in traditional drug therapy for ischemic stroke. Furthermore, we presented an in-depth analysis of the role of polymeric nanoparticles as effective carriers in various targeted drug delivery systems, specifically tailored for ischemic stroke treatment. This comprehensive examination laid a critical foundation for the future development of innovative intracerebral therapeutic strategies, heralding a new era in stroke management.

## Pathological mechanisms of cerebral ischemic stroke

**Fig. 1:**
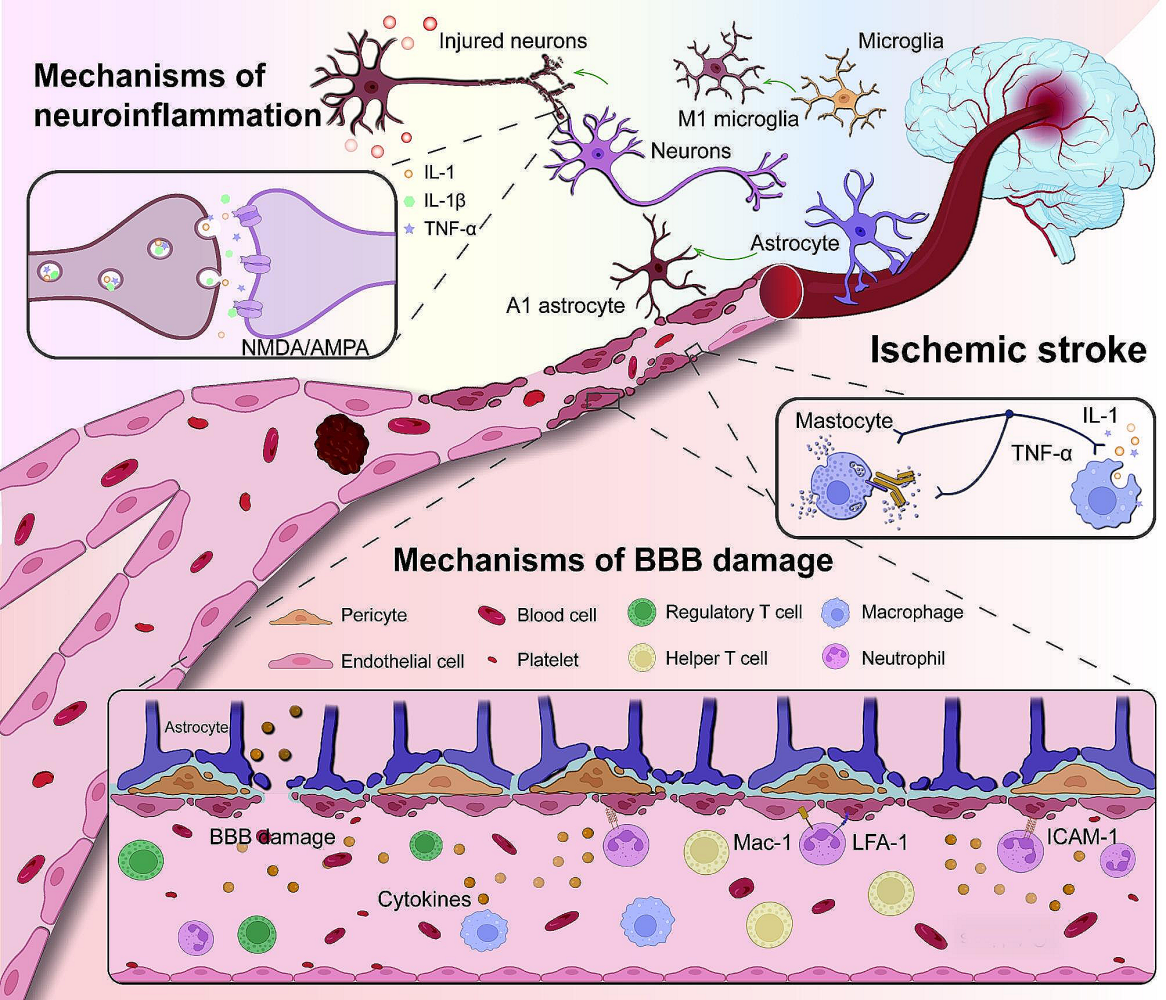
Schematic diagram of the pathogenesis of ischemic stroke, including the mechanism of neuroinflammatory response and the mechanism of BBB damage. The mechanisms of neuroinflammation: NMDA/AMPA pathway activation, inflammatory cytokine release (IL-1, IL-1β, TNF-α), mast cell degranulation, astrocyte and macrophage polarization, neuronal damage occur. The mechanisms of BBB damage: Damage to endothelial cells and breaks in membrane tight junctions

In the initial phase of ischemic stroke, a critical event is the translocation of P-selectin to the surface of platelets and endothelial cells within the vascular lumen. This translocation is a precursor to the aggregation of platelets and leukocytes, a process intricately mediated by key adhesion molecules. Subsequently, the adhesion of these cellular aggregates is facilitated through the interaction of intercellular cell adhesion molecule-1 (ICAM-1), lymphocyte function-associated antigen-1 (LFA-1), and Macrophage-1 (Mac-1) [21–23]. Concurrently, neuronal tissue in the cerebral ischemic environment undergoes a cascade of pathophysiological alterations, rendering it particularly vulnerable to deficits in oxygen, glucose, and other vital nutrients. These changes and their implications were systematically depicted in Fig. 1, providing a comprehensive overview of the ischemic cascade.

Initially, a reduction in cerebral blood volume instigates a series of detrimental effects, including neuronal injury, metabolic imbalances, and inflammatory responses characterized by acidosis and cellular edema [24–26]. This scenario is further exacerbated by changes in cellular ion concentrations, leading to increased depolarization of neurons and astrocytes. Accompanying this is the abnormal accumulation of glutamate-activated receptors, specifically N-methyl-D-aspartate (NMDA) and alpha-amino-3-hydroxy-5-methylisoxazole-4-propionic acid (AMPA) receptors, which precipitate an atypical influx of calcium ions [27, 28]. Concurrently, the hydrolysis of neuron-activated enzymes results in the production of harmful substances, such as free radicals. This enzymatic activity culminates in an increased concentration of diverse inflammatory cytokines (TNF-α, IL-1, and TGF-β). These cytokines intensify the ischemic condition and contribute to the disruption of the BBB, through a cascade of oxidative stress and inflammatory responses [29–32]. Moreover, mast cells and perivascular macrophages secrete cytokines (TNF and IL-1), further facilitating the migration of inflammatory cells across the vascular wall, thus compounding the inflammatory milieu within the ischemic cerebral tissue [33].

In the disruption of the BBB, two pivotal stages are delineated: the early cerebral ischemia phase and the subsequent post-ischemic reperfusion phase. Initially, cerebral ischemia induces a proliferation of endothelial cell vesicles, facilitating abnormal molecular transference into the brain via augmented endocytosis and transcytosis [34]. This phase is further characterized by the compromise of tight junctions within endothelial cells, a crucial factor in elevating the BBB’s permeability, thereby predisposing it to additional injury during the ensuing reperfusion [35, 36]. The reperfusion phase, following cerebral ischemia, marks a further decline in BBB integrity as restored blood flow leads to increased oxidative stress and heightened inflammatory responses. These changes are driven by the abrupt restoration of blood circulation and are marked by the infiltration of inflammatory cells, production of reactive oxygen species, and release of cytokines. Collectively, these factors exacerbate the structural and functional degradation of the BBB. Ultimately, sustained ischemia and anaerobic glycolysis contribute to the accumulation of lactate in neural tissues, concurrently diminishing ATP levels and pH, which culminates in irreversible neurological damage [37, 38]. It is noteworthy that cerebral ischemia-induced alterations in the BBB significantly impact pharmacotherapy. The compromised BBB facilitates increased drug permeability, allowing greater drug access to the brain, which can be beneficial for treatment, yet simultaneously poses risks of undesired side effects and toxicity [38]. Moreover, alterations in the BBB can lead to uneven drug distribution within the brain, potentially affecting the drug’s metabolism and clearance rates, thereby reducing therapeutic efficacy or increasing adverse effects. Damage to the BBB may also exacerbate inflammatory and oxidative stress responses, further influencing drug effectiveness and safety [39, 40]. Consequently, in the development of drugs for neurological conditions, the drug’s ability to penetrate the BBB and how this ability changes with the state of the BBB must be considered. It also necessitates close monitoring of drug effects and potential side effects during treatment [41].

## Conventional drug treatment strategies

Traditional treatment methods for ischemic stroke, as outlined in Table 1, consist of pharmacological and surgical interventions [42]. For instance, aspirin at a dose of 300 mg daily for up to 14 days, when initiated within 48 h, has been shown to enhance prognostic mortality, although its advantage is relatively minor [43, 44]. Notably, tPA remains the globally acknowledged treatment for ischemic stroke, with a critical administration window of 4.5 h post-stroke onset.

However, current treatment strategies for ischemic stroke confront several challenges [45]. These include the restrictive nature of the BBB [46], which significantly limits drug delivery to targeted sites. Also, issues such as poor drug stability and a short half-life within the body compound the difficulty in controlling drug dosage and timing of administration to ischemic sites. Additionally, the lack of targeted drug delivery mechanisms exacerbates systemic reactions and elevates the risk of hemorrhagic complications. Furthermore, inherent toxicity of certain drugs can cause secondary neuronal damage, and reperfusion injury is a persistent concern [47, 48]. In summary, conventional ischemic stroke treatments have multiple limitations and often result in numerous drug-related side effects. Therefore, addressing and overcoming the drawbacks of intracerebral drug administration is crucial in advancing therapeutic approaches [49].

**Table 1:** Conventional treatment strategies for ischemic stroke

Type	Category	Drugs	Refs.
Medication	Anticoagulant drugs	Warfarin Heparin	[50]
	Antiplatelet drugs	Aspirin Clopidogrel	[51]
	Anticoagulant	tPA	[52]
	Thrombolytics	Urokinase Streptokinase	[53]
	Neuroprotector	Nitric oxide antagonists Glutamate blockers Calcium chelators	[54]
	Renin-angiotensin-aldosterone system inhibitors	Clofentezan, Valsartan Eprosartan Temisartan Perindopril	[55]
	Cholesterol reducers	Rosuvastatin Simvastatin	[55]
Surgery	Mechanical embolization		[56]
	Stent placement		[56]
	Neurosurgery		[55]

## Polymer nanocarrier delivery system therapeutic strategies

Nanocarrier delivery systems are nanoscale carriers or technologies used to deliver drugs or therapeutic substances into the body [57]. These systems are intricately designed to augment drug bioavailability, mitigate adverse drug reactions, and enhance treatment precision [58, 59]. A key feature of nanocarriers is their small size, which offers an increased surface area, thereby enhancing interaction with biological tissues. This results in improved drug bioavailability and therapeutic efficacy. Additionally, most nanocarriers, especially those that are chemically modified, exhibit high encapsulation efficiency and loading capacity. Such attributes are instrumental in protecting the drug from premature denaturation due to environmental interferences. Furthermore, these modified systems demonstrate notable transmembrane permeability, allowing drugs to reach deeper tissues, which in turn augments drug solubility, absorption, and bioavailability. Another salient feature of many nanocarrier systems is the improvement in pharmacokinetic properties, including enhanced drug stability and extended half-life. Significantly, systems modified with various chemical alterations possess targeting and controlled-release characteristics. These modifications enable the controlled release of drugs and targeted delivery to specific tissues or regions via surface chemical alteration sites. Moreover, certain systems enhance patient compliance by maintaining effective drug concentrations in target tissues and reducing the frequency of dosing required for patients [60–63]. In stroke treatment, the ultimate objective of nanomaterial delivery as a drug carrier is to safely and stably administer the appropriate concentration and dose of the drug to the ischemic lesion within the effective therapeutic time window while minimizing neuronal cell damage and avoiding inflammatory cascade reactions.

This article primarily focuses on the application of polymer nanocarriers in targeted drug therapy for stroke [64, 65]. These systems can be broadly categorized into two groups for clarity and specificity in their presentation. The first group, conventional systems, such as polymer nanoparticles and polymeric micelles, which are well-established in their therapeutic applications, particularly in ischemic stroke therapeutics. The second group, advanced systems, which include biolipid-coated polymer nanocarriers, nucleic acid self-assembling nanocarriers, and other atypical polymer nanocarrier systems. These advanced systems are distinguished by their complex structures and specialized functions, reflecting the latest advancements in nanocarrier technology and offering new avenues for medical research and application. Favored for their enhanced stability and biocompatibility, these carriers can be developed for potential diverse applications by chemically altering their hydrophilic-lipophilic balance, charge, and physical structure [66]. Moreover, compared to other types of nanocarriers, polymer nanocarriers offer a broader range of control over the duration of drug delivery, enabling effective regulation of drug release and degradation.

### Polymer nanoparticles

Polymer nanoparticles generally range in size from 10 nm to 1,000 nm and can be divided into two broad categories based on their origin: natural polymer nanoparticles and synthetic polymer nanoparticles [67]. Natural polymer nanoparticles refer to some biological macromolecules derived from nature, which can be processed into nanoparticles through specific physical or chemical methods. They are characterized by good biocompatibility and biodegradability and are commonly used in drug delivery systems [68, 69].

Synthetic polymer nanoparticles are fabricated through chemical synthesis processes in controlled laboratory settings [70]. Their structural and chemical properties can be meticulously tailored during the synthesis process to align with specific application needs. Typically, these nanoparticles are part of a drug delivery system, which is formed by emulsifying polymeric materials. This includes polymers like polyethylene glycol (PEG) [71–76], polylactic acid (PLA), and polylactic-co-glycolic acid (PLGA) [77–81]. These systems act as self-catalytic regulators of the BBB via internal encapsulation mechanisms. They are capable of achieving the controlled release and targeted delivery of insoluble drugs, thus mitigating their toxicity and side effects when administered in vivo.

#### Natural polymer nanoparticles

Natural polysaccharides are increasingly gaining prominence in the field of biomaterials due to their excellent non-toxicity, high biocompatibility, and desirable biodegradability. Through the process of amination, a range of cationic polysaccharides has been designed and synthesized to serve as efficient drug delivery systems. Examples include cationic amylopectin [82–84], chitosan [85, 86], dextran [87, 88] and fucose [89–91]. These natural polysaccharide carriers offer distinct advantages over other polymers, such as enhanced cell internalization facilitated by sugar recognition receptors on the cell surface, low immunogenicity, and high solubility. Consequently, an increasing number of studies are focusing on developing new polysaccharide polymer nano-delivery systems specifically for the treatment of ischemic stroke.

**Fig. 2:**
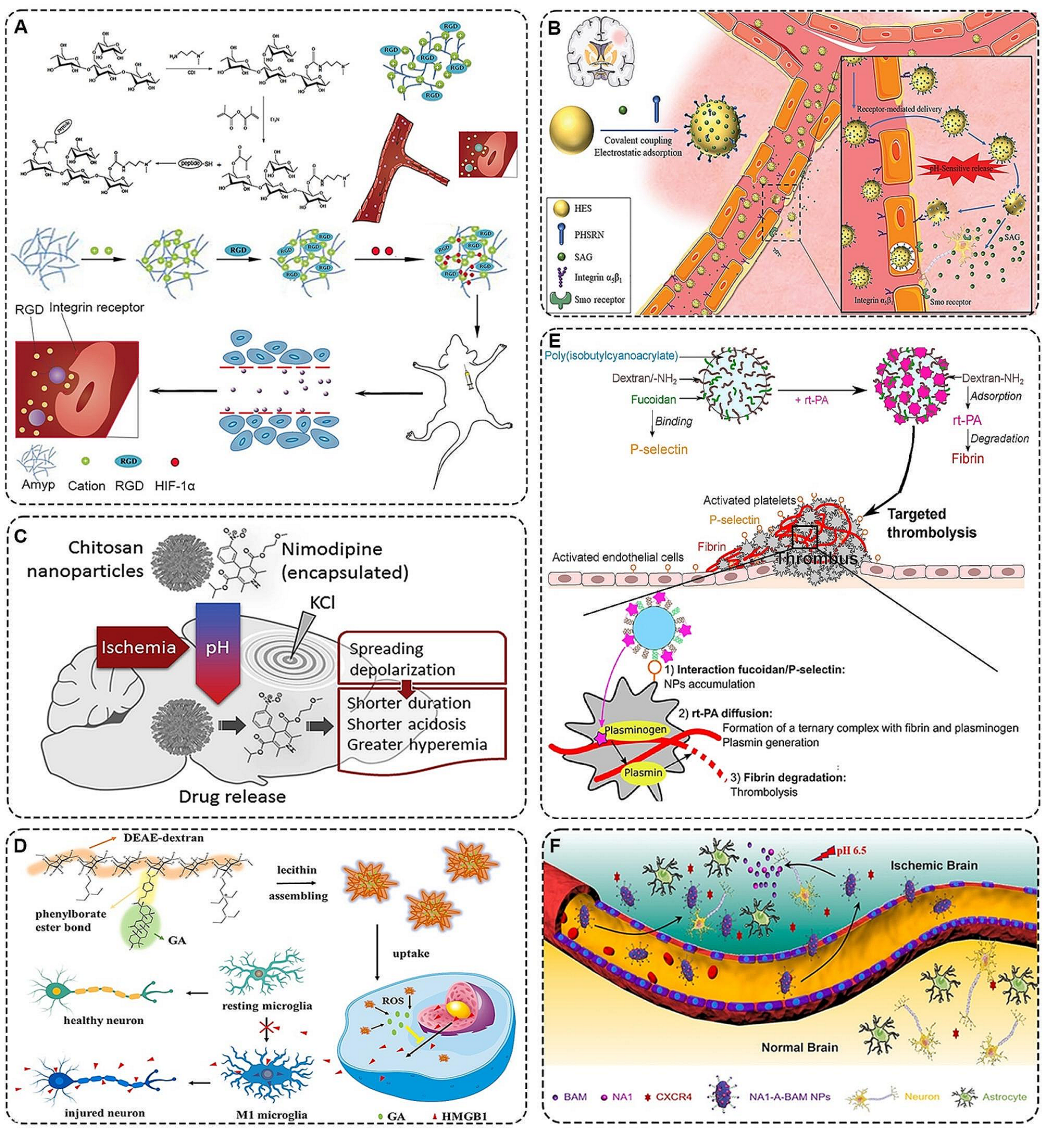
**A** Schematic illustration of the fabrication of RGD-DMAPA-Amyp/HIF-1α-AA and its mechanism in the treatment of cerebral ischemia [83]. Copyright 2019, American Chemical Society. **B** Schematic structure of SAG@PHSRN-HES and its role in active cerebrovascular targeting and pH sensitivity for the treatment of ischemic stroke [84]. Copyright 2021, Wiley-VCH GmbH. **C** PH-responsive chitosan nanoparticles releasing nimodipine directly to the brain surface exerted vasodilatory effects [86]. Copyright 2019, Elsevier Ltd. **D** ROS-responsive drug-conjugated nanoparticles inhibit HMGB1 and modulate microglia polarization [87]. Copyright 2022, Elsevier B.V. **E** Interaction of nanoparticles with thrombus [89]. (1) Nanoparticles specifically aggregate on thrombus via fucoidan/P-selectin interaction. (2) rt-PA can diffuse within the fibrin network and forms a ternary complex with fibrinogen on fibrin. This initiates the formation of a plasmin. (3) Plasmin produces fibrinolysis. Copyright 2017, Elsevier Ltd. **F** Schematic representation of the application of NA1-A-BAM NPs to stroke therapy [93]. Copyright 2022, Elsevier B.V

Branched starch is a hyperbranched polysaccharide whose hydroxyl groups can be used for simple chemical modifications, such as amination. Recent research has highlighted that aminated derivatives of cationic amylopectin serve as effective non-viral gene delivery vectors with high transfection efficiency [82]. For example, Zhou et al. constructed a target gene vector hyperbranched cationic polymer (DMAPA-Amyp) modified with ligand RGD (Fig. 2A). Experimental data demonstrated that the RGD-containing nano-carrier could penetrate vascular endothelial cells in the infarcted area by binding to over-expressed integrins. In vivo studies confirmed that the RGD-DMAPA-Amyp, loaded with a mutant hypoxia-inducible factor-1α (HIF-1α), effectively treated cerebral infarction by promoting neurological function recovery [83]. In addition, amylopectin hydroxyethylstarch (HES) is clinically used to expand blood volume because it can delay decomposition and elimination in the blood and significantly prolong its intravascular residence time. Utilizing this property, Yang’s team prepared a smoothened agonist (SAG) @ Pro-His-Ser-Arg-Asn (PHSRN)-HES with a dual targeting function (Fig. 2B). On the one hand, the nanoparticles selectively aggregated in the ischemic foci through the interaction of the over-inhibited integrin 5-amino-1 with the PHSRN peptides attached to the HES surface. On the other hand, the acidic microenvironment of cerebral ischemia induced the release of SAG from nano-carriers. SAG activated Smo receptors on the cell membrane to enhance angiogenesis and neuroplasticity [84].

Chitosan, a readily available natural polysaccharide, has been studied for its unique properties. Jan et al. confirmed that when chitosan is aminated, it exhibits polyelectrolyte properties with pH-responsive solubility [85]. They also demonstrated that nimodipine, a neural tissue drug, when associated with pH-responsive chitosan nanoparticles, effectively released in ischemic or spreading depression-induced tissue acidosis conditions (Fig. 2C). This approach could significantly enhance ischemic stroke treatment by dilating blood vessels, protecting neural tissue, and preventing depolarization spread to the penumbra [86].

Dextran, a water-soluble polysaccharide produced by extracellular bacteria, is extensively applied in clinical settings due to its capabilities in preventing thrombosis, expanding plasma volume, and enhancing peripheral blood flow [88]. Based on these advantages of dextran, Jin et al. designed ROS-responsive 18β-glycyrrhetinic acid (GA)-conjugated diethylaminoethylen (DEAE)-dextran nanoparticles (DGA) (Fig. 2D). This polymer effectively delayed stroke progression, reduced infarct volume, and promoted nerve regeneration by inhibiting HMGB1 translocation and microglial M1 polarization [87].

Fucose is a type of monosaccharide often found in sulfated polysaccharides derived from seaweed and gums [90]. Chauvierre et al.‘s study highlighted fucose’s strong affinity for P-selectin expressed by activated platelets in thrombi (Fig. 2E). The polymer accumulated specifically and released rtPA at thrombi through fucoidan/P-selectin interaction, facilitating fibrinolysis [89]. Based on this study, the team also constructed a new fucoidan functionalized dextran subparticles (SPS) through green chemical methods. In vivo results showed that rtPA-associated Fuco-SPs minimized brain damage due to their better thrombolytic effect and faster revascularization rate [91].

The latest research suggested that many natural nanomaterials could also be used as drug delivery carriers by forming nanoparticles. For example, Deng et al. isolated a natural nanoparticle compound betulinic acid (BA) from medicinal plants. They utilized it as a drug carrier of glibenclamide to achieve the combined treatment of anti-edema and antioxidation in ischemic stroke [92]. The team’s latest study chemically converted betulinic acid into the acidic microenvironment of betulin (BAM) according to brain defect areas [93]. The results showed that AMD3100 conjugated betulinicamine (A-BAM) could deliver NA1 to ischemic brain regions more efficiently and protect neurons from NMDA receptor-mediated excitotoxicity in acidic microenvironment (Fig. 2F).

#### Synthetic polymer nanoparticles

**Fig. 3:**
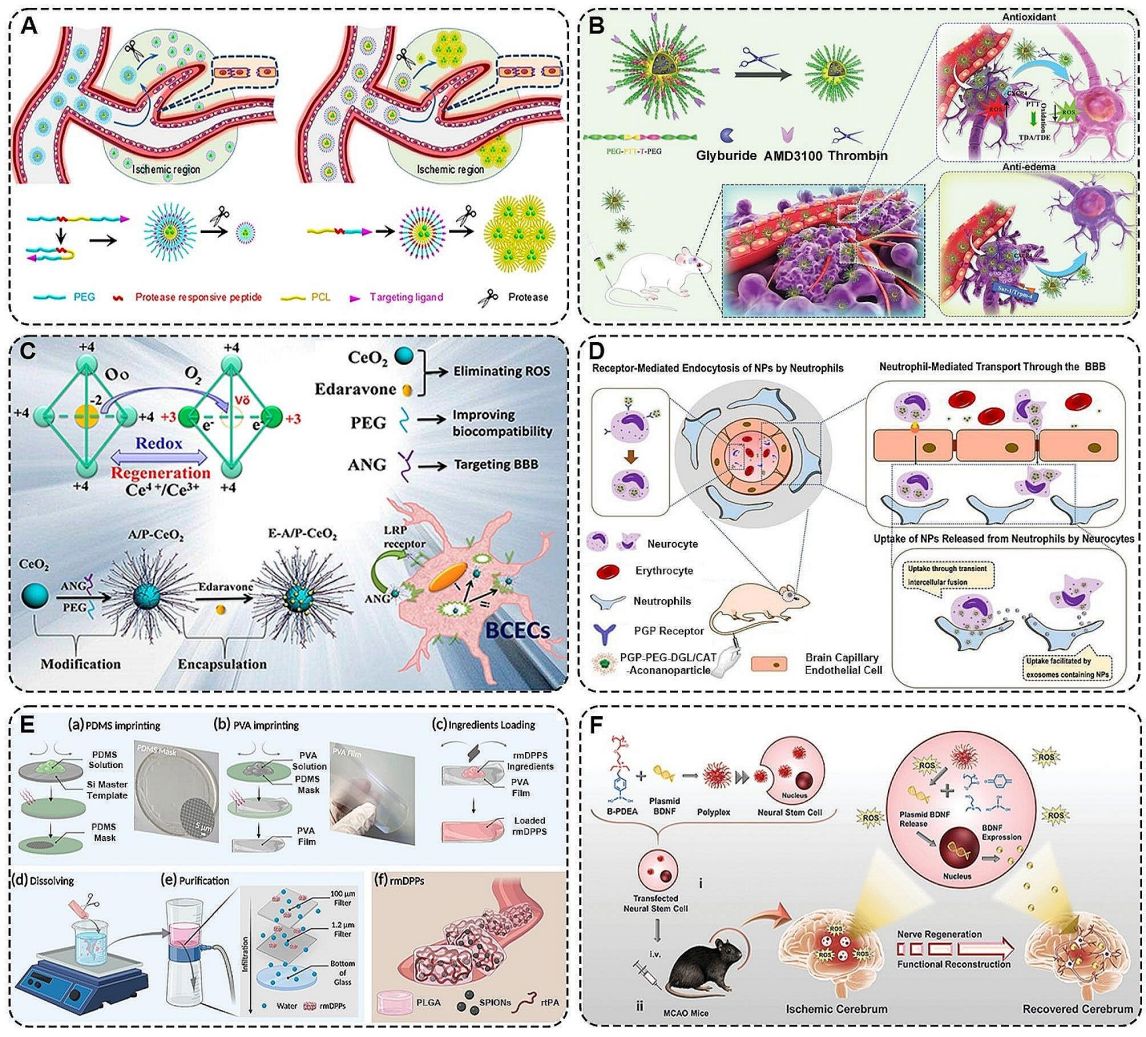
**A** Schematic representation of the ability of micellar nanoparticles to change their size in response to the ischemic microenvironment (contraction and expansion) in the presence of different proteases [71]. Copyright 2018, American Chemical Society. **B** Schematic illustration of PEG-PTT-T-PEG block copolymer self-assembly constructs and size reduction in the presence of thrombin [72]. ASPTT has brain-targeting, ROS-targeting, and anti-edema effects. Copyright 2022, Wiley-VCH GmbH. **C** Schematic depiction of the construction of E-A/P-CeO2 and the main functions of each part [73]. (i) BBB protection through receptor-mediated endocytosis. (ii) Treatment of stroke by transcytosis across the BBB. Copyright 2018, American Chemical Society. **D** Schematic diagram of the PGP-PEG-DGL/CAT-Aco NPs system utilizing neutrophil-mediated brain targeting for the treatment of ischemic stroke [74]. Copyright 2017, Ivyspring International Publisher. **E** A scheme showing the construction process of rmDPPs and its morphologic structure in the vasculature [81]. Copyright 2022, Springer Nature. **F** Schematic representation of the construction of B-PDEA/BDNF plasmid (pBDNF) and the therapeutic effect of stroke [97]. Copyright 2019, Wiley-VCH Verlag GmbH & Co. KGaA, Weinheim

PEG is a polymer extensively utilized in constructing nanoparticles for stroke treatment, owing to its exceptional biocompatibility, monodispersity, and ability to prolong drug half-life in the bloodstream. Gao et al. developed polymer nanoparticles comprising PEG, enzyme-cleavable peptides, and poly(ε-caprolactone) (PCL), with AMD3100-conjugated size-shrinkable nanoparticles (ASNPs) for ischemic brain tissue targeting (Fig. 3A). These nanoparticles adapted their size in response to abundant enzymes in the ischemic microenvironment, thereby not only enhancing BBB permeability but also improving the delivery of the anti-edema drug glibenclamide to the brain [71]. Additionally, the team developed another delivery system, AMD3100-conjugated PEG-poly(2-methylene-thiodiethylene-3-thiodipropionate) (PTT)-T-PEG glibenclamide system (Fig. 3B), incorporating PTT for ROS responsiveness and thrombin-stimulated ischemic microenvironment response. This system exhibited high brain permeability and antioxidant activity, effectively releasing glibenclamide [72]. In addition, Bao et al. designed edaravone-carried and PEG/Angiopep-2(ANG)-conjugated ceria nanoparticles (E-A/P-CeO_2_) which could efficiently treat stroke. In Fig. 3C, it was illustrated that CeO_2_ and Edaravone enhance biocompatibility and target the BBB through distinct mechanisms: CeO_2_ scavenged ROS, contributing to its therapeutic function, while PEG improved stability and biocompatibility, and ANG facilitated targeted delivery to the BBB [73]. Furthermore, the modifiability of polymer nanoparticle surfaces allows for targeted migration and treatment of ischemic foci. Zhang et al. fabricated a system with an endogenous high-affinity ligand acetyl Pro-Gly-Pro (Ac-PGP) for neutrophil anchoring, using dendritic graft poly L-lysine (DGL) as a carrier and introducing cis-aconitic anhydride-modified catalase (CAT) for increased charge density (Fig. 3D) [74]. Additionally, the hollow-structured MnO_2_ (H-MnO_2_)-PEG [75], cationic bovine serum albumin-conjugated tanshinone IIA PEG-conjugated nanoparticles (CBSA-PEG-TIIA-NPs) [76] have shown efficacy in penetrating the BBB, releasing their drug payload, and improving ischemic stroke outcomes through specialized drug delivery system design.

PLGA, a copolymer synthesized from glycolic acid and lactic acid, is renowned for its excellent biodegradability, biocompatibility, and adjustable properties. It has been a focus of many studies aiming to create enduring drug release systems for ischemic stroke, leveraging PLGA NPs that can traverse the BBB and localize in ischemic regions [94]. For example, Han et al. built a PLGA drug delivery system using chloramphenicol (CTX) as a surface-targeting ligand, with internal encapsulation of lexiscan (LEX) for targeted delivery [77]. Similarly, the use of PLGA to encapsulate antioxidants including CAT and superoxide dismutase (SOD) could be utilized in the treatment of ischemic strokes [78]. Additionally, researchers constructed peptide nucleic acid (PNAs) and phosphorothioates (PS) @ PLGA by double emulsion solvent evaporation technology [79]. The results indicated that PLGA could encapsulate the optimal amount of anti-miR-141-3p based on PS and PNA (miRNA significantly up-regulated after stroke), and significantly reduce miR-141-3p levels and infarct volume after in vivo release. Furthermore, to achieve more accurate drug delivery in vivo, PLGA could also combine with magnetic nanoparticles (MNP) to form PLGA magnetic nanocomposites. For example, Chen et al. designed a rtPA-embedded PLGA MNP (PMNP) that offered a dual-targeted thrombolytic therapy strategy [80]. The results showed that PMNP could precisely deliver rtPA to specific locations in the body through the guidance of an external magnetic field. Meanwhile, the biodegradable nature of PLGA allowed the slow release of rtPA in the target area, effectively reducing the therapeutic dose of rtPA and lowering the risk of bleeding. What’s more, Cui et al. prepared PLGA loaded with rtPA and superparamagnetic iron oxide nanoparticles (SPIONs) (rmDPPs) using a top-down approach [81]. Specifically, they used semi-permanent polydimethylsiloxane (PDMS) molds as master templates to synthesize and mold disposable polyvinyl alcohol (PVA) films, which were then collected by filtration and purification to obtain rmDPPs (Fig. 3E). Experimental results demonstrated that rmDPPs could target the desired sites using magnetic fields and release rtPA abruptly upon acoustic stimulation. In vivo results showed that delayed treatment was also effective without hemorrhagic transformation.

Recent studies revealed that neural stem cells (NSCs) were capable of actively nesting and concentrating in damaged brain tissue areas after ischemic stroke [95, 96]. They could regenerate and differentiate into damaged cell phenotypes to repair neurological damage. Nonetheless, the efficacy of NSCs is constrained by the adverse conditions of the ischemic microenvironment. To overcome this problem, Jiang et al. fabricated ROS-responsive charge-reversal poly[(2-acryloyl) ethyl (p-boronic acid benzyl) diethylammonium bromide] (B-PDEA) polymer carrier (Fig. 3F). Notably, ischemic stroke mice receiving these transfected NSCs via tail vein injection exhibited significantly elevated levels of BDNF expression and remarkably improved survival rates in vivo [97]. This breakthrough highlighted the potential of advanced polymer carriers in enhancing the therapeutic effectiveness of NSC-based treatments for ischemic stroke.

### Polymer micelles

**Fig. 4:**
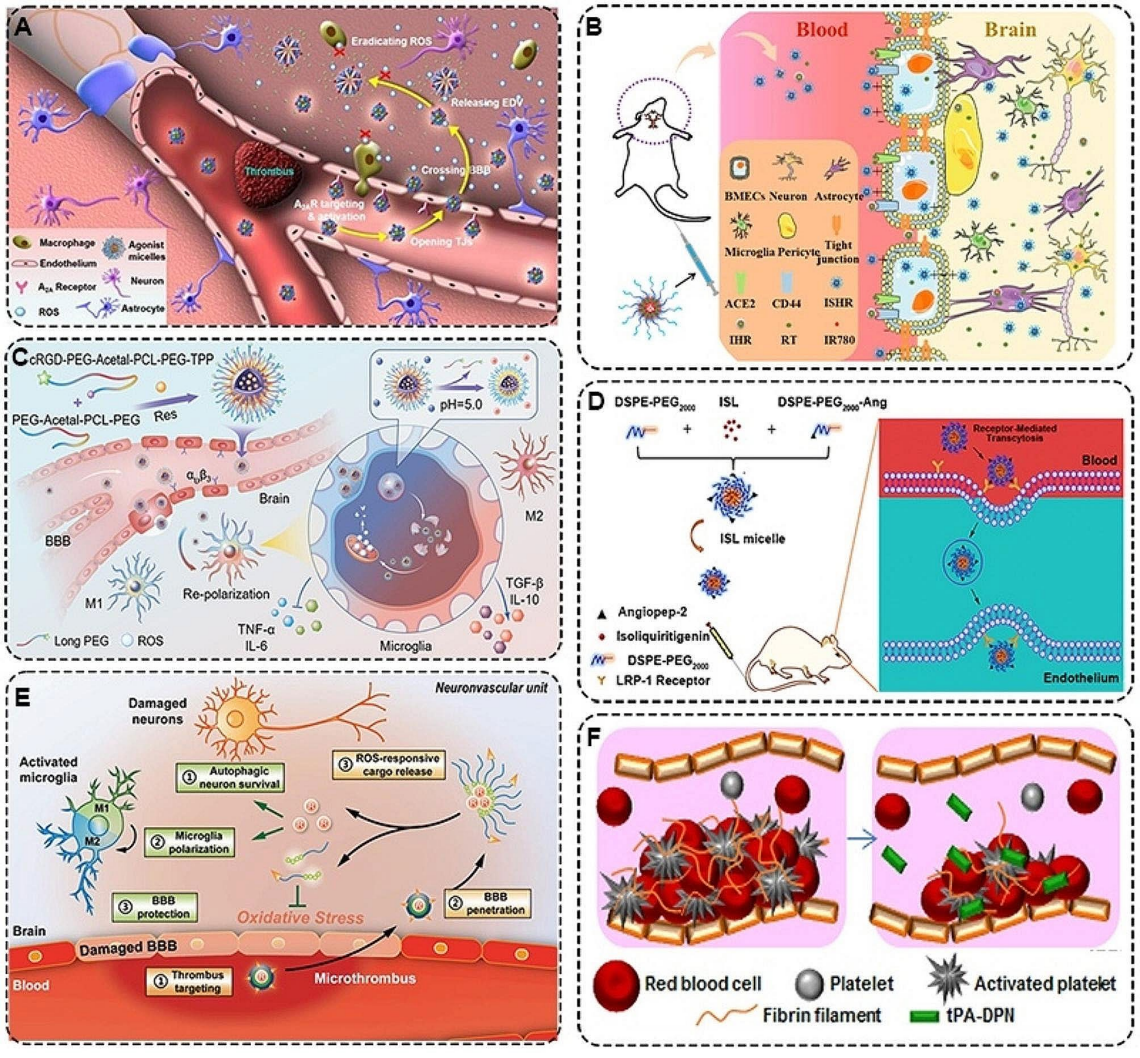
**A** Schematic diagram of agonist micelles rescuing cerebral ischemic tissue [99]. Copyright 2017, Ivyspring International Publisher. **B** Schematic depiction of SHR micelles crossing the BBB [101]. Copyright 2023, American Chemical Society. **C** The stepwise targeting nanoplatform can effectively mitigate oxidative stress and inflammation by enhancing resveratrol delivery to microglia mitochondria and reverse microglia phenotype by scavenging ROS [102]. Copyright 2023, American Chemical Society. **D** Schematic preparation of ISL micelles and their LRP-1 receptor-mediated BBB permeation mechanism [103]. Copyright 2022, Dove Medical Press Limited. **E** Illustration of CPLB/RAPA micelle formation and regulation of the neurovascular unit in the ischemic brain [105]. Copyright 2019, WILEY-VCH Verlag GmbH & Co. KGaA, Weinheim. **F** Schematic representation of the composition of tPA-DPNs and intravascular thrombolysis [106]. Copyright 2018, American Chemical Society

Polymeric micelles, as a kind of simple nanostructures are mainly formed by self-assembly of amphiphilic block copolymers, which have the advantages of lower toxicity, higher drug loading, and stronger stability. Their hydrophobic core can encapsulate hydrophobic drugs or other small molecules, while the hydrophilic shell enables the micelles to be stabilized in an aqueous environment [98]. For example, edaravone (EDV) is a hydrophobic drug that effectively scavenges ROS production by microglia as well as infiltrating inflammatory cells. However, its short half-life and insufficient uptake greatly diminish its therapeutic effect. With this in mind, Jin et al. developed the first EDV delivery system that exerted neuroprotective effects by actively modulating BBB permeability to eliminate ROS in the brain (Fig. 4A). This agonistic micelle (EDV-AM) was able to open the primary structure regulating BBB permeability (tight junction TJ) and specifically deliver EDV to the site of cerebral ischemia [99].

In recent years, various studies have highlighted the potent antioxidant and anti-inflammatory properties of natural small-molecule compounds, suggesting their potential in ischemic stroke treatment. However, challenges such as low bioavailability, short half-life, and difficulty in crossing the BBB often limit their therapeutic effectiveness. To address these issues, researchers have constructed numerous polymeric micelles incorporating natural small molecule compounds. For instance, Li et al. pointed out that triblock copolymer nanomicelles loaded with curcumin could improve the inflammatory response in ischemic stroke by inhibiting the NF-κB pathway [100]. Moreover, taking into account that ischemia and hypoxia in stroke create an acidic environment and the overexpression of CD44 and hyaluronidase 1, Zhao et al. fabricated a triple-targeted SS-31-hyaluronicacid-rutin polymeric micelle (SHR) (Fig. 4B). On the one hand, the micelle utilized CD44-mediated endocytosis and the targeting effect of the synthetic mitochondrial peptide SS-31 to jointly achieve the effect of drug penetration through the BBB and targeting the damaged brain site. On the other hand, the acidic environment and hyaluronidase 1 promoted the sustained release of rutin in the ischemic brain region by degrading HA, which in turn activated ACE2 and TFEB signaling for synergistic brain tissue repair [101]. Similarly, Wang et al. constructed cyclo(Arg-Gly-Asp-DTyr-Lys) peptide (cRGD)/ Triphenylphosphine (TPP) nanomicelles to load and release the antioxidant resveratrol (Res) (Fig. 4C). cRGD/TPP@ Res was able to efficiently target the release of Res in the acidic stroke microenvironment, thereby reducing oxidative stress and neuro-inflammationvia promoting microglia phenotypic transformation (M1-M2) [102]. In addition, Song et al. prepared the brain-targeting peptide Angiopep-2-modified DSPE-PEG2000 (ISL-M) loaded with isoliquiritigenin (ISL) [103]. This platform enhanced ISL accumulation in the ischemic brain region via LRP-1 receptor-mediated endocytosis across the BBB. The effective release of ISL ameliorated neuronal damage and reverses behavioral deficits induced by ischemic stroke (Fig. 4D).

Stroke research has identified that mammalian target of rapamycin (mTOR) activity is often elevated during a stroke event, and that inhibiting this activity can promote beneficial autophagy, thereby enhancing neuronal survival by removing damaged organelles [104]. Leveraging this insight, researchers developed a polymeric micellar system, CREKA-PEG-LysB, designed for neurovascular targeting and ROS responsiveness. This system facilitated the delivery of the mTOR inhibitor rapamycin directly to brain tissue (Fig. 4E). The CREKA-PEG-LysB polymer incorporated a multifaceted approach to ischemic stroke treatment. It combined vascular and neuroprotection strategies, including antioxidant actions, protection through autophagic neuron survival, and promotion of a microglial phenotypic shift from M1 to M2 [105]. This comprehensive strategy exemplified the innovative approaches being developed in the field of stroke therapeutics, particularly those targeting the complex molecular pathways involved in stroke pathology.

Researchers have been referencing cellular structures and cytoplasmic matrix compositions in their design of polymeric micelles to more accurately replicate conditions found in living organisms, aiming to enhance the treatment of ischemic stroke. For instance, they have combined the clinical formulation of tPA with the porous structure of soft discoidal polymeric nanoconstructs (tPA-DPNs) [106]. Compared to tPA alone, this polymer displayed increased stability and a more efficient thrombolytic capacity. This was attributed to its red blood cell-like shape and deformability, which contributed to effective circulatory characteristics and the accumulation at blood clot sites (Fig. 4F). Moreover, the cytoplasmic matrix component Reelin, known for effectively regulating neuronal migration and inducing vascular sprouting, has been noted to delay the progression of ischemic stroke. As an example of this application, Shabani et al. prepared PLGA-PEG nanomicelles loaded with Reelin. These nanomicelles have been shown to promote neuronal differentiation, the growth of NSCs, and angiogenesis following ischemic injury [107]. This approach showed the potential of leveraging intrinsic cellular components and mechanisms in developing advanced therapeutic strategies for ischemic stroke treatment.

### Biofilm-coated polymer nanocarrier delivery system

**Fig. 5:**
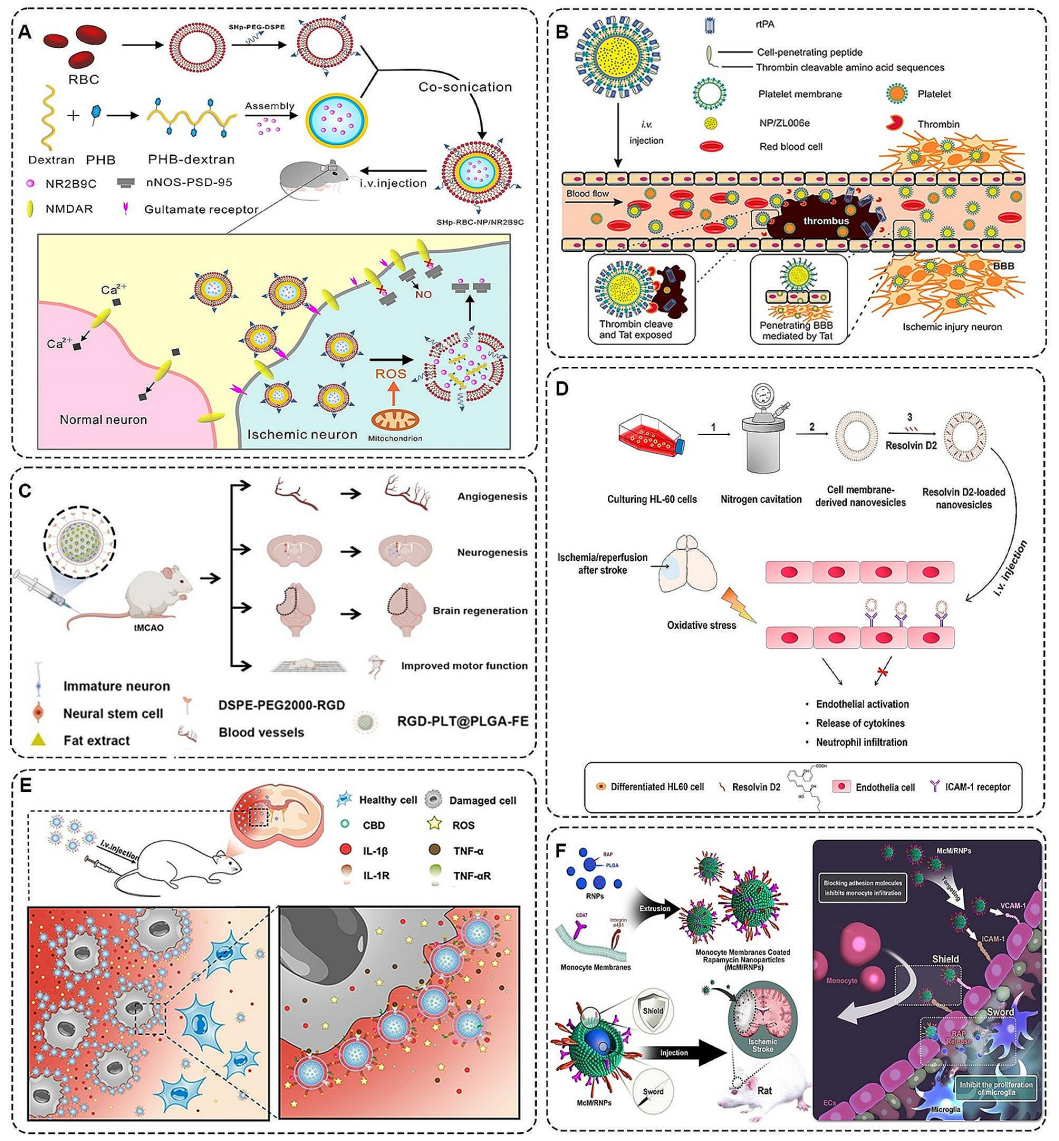
**A** Schematic representation of the composition of SHp-RBC-NP/NR2B9C [112]. SHp-RBC-NP/NR2B9C has a targeted neuroprotective effect on ischemic brain regions mediated mainly through the model stroke homing peptide. Copyright 2023, Wiley-VCH GmbH. **B** A scheme showing the composition of tP-NP-rtPA/ZL006e [114]. tP-NP-rtPA/ZL006e has the ability to target the release of rtPA under thrombin activation and transport ZL006e across the BBB. Copyright 2019, American Chemical Society. **C** Schematic illustration of the composition of RGD-PLT@PLGA-FE [115]. The active targeting ability and neuroprotective effects of RGD-PLT@PLGA-FE on ischemic stroke were validated in a mouse model. Copyright 2022, Springer Nature. **D** Schematic design structure of RvD2-HVs and their specific binding to brain endothelial cells to exert anti-inflammatory effects in the treatment of ischemic stroke [116]. Copyright 2019, American Chemical Society. **E** Morphological structure of LA-NM-NP/CBD and anti-inflammatory effects of targeting ischemic hemispheric regions [117]. Copyright 2022, American Chemical Society. **F** Schematic representation of the composition of McM/RNPs and their anti-inflammatory effects on active inhibition of macrophage proliferation [118]. Copyright 2021, Springer Nature

The membrane structure of cells is crucial in delineating internal and external environments, facilitating substance exchange, and enabling signal transmission [108]. In recent advancements, researchers have developed complex structured novel drug delivery systems. These systems involved encapsulating therapeutic drugs or small molecules, which were then integrated or attached to recognition ligands on the surface of cell membranes, further enhanced by modified biofilms [109–111]. Compared to simple polymeric micelles, this approach synergized the natural properties of biofilms with the advantages of nanotechnology, resulting in superior biocompatibility, precise drug targeting, and sustained drug release capabilities. Among them, mimicking red cell membranes as nanoparticle structures was a promising strategy for the treatment of stroke because of better immune escape and longer drug release time in the brain. For instance, Lv et al. developed a ROS-responsive core-shell nanocarrier for targeted delivery of the neuroprotective agent NR2B9C. The carrier extended the duration of the nanocarrier in the blood circulation by mimicking the properties of red blood cells [112]. The carrier was composed of a boric acid-modified dextran polymer core and a shell made of erythrocyte membrane surface-affixed with stroke homing peptide (SHp) (SHp-RBA-NP) (Fig. 5A). This design selectively disrupted the interaction between the N-methyl-D-aspartate receptor (NMDAR) and postsynaptic density protein (PSD-95), facilitating neuroprotective therapy for ischemic stroke.

During ischemic stroke, red blood cell rupture often occurs at cerebrovascular injury sites, accompanied by platelet adhesion, activation, and aggregation. These processes are major contributors to intravascular thrombosis [22, 23, 71]. The researchers used the characteristic of platelet aggregation in the ischemic microenvironment to construct polymer nano-carriers coated with platelet membranes. For example, Xu et al. utilized platelet membranes affixed with rt-PA wrapped around the surface of polymer nanoparticles. They constructed a novel biomimetic nanocarrier PNP-PA with long-lasting drug release and thrombolytic targeting [113]. Based on this, the team also developed a core-shell polymer nanocarrier capable of sequentially targeting the delivery of thrombolytic and neuroprotective agents (tP-NP-rtPA/ZL006e), which they named “nanoplatelets” (Fig. 5B). The team covered platelet membranes with BA-conjugated rt-PA on the surface of acetal-modified dextran (m-dextran) polymer nanoparticles loaded with ZL006e, a neuroprotectant that selectively inhibits ischemia-induced PSD-95/nNOS coupling. They combined biomimetic and bioresponsive technologies to achieve stimulated release of rtPA and intracerebral delivery of neuroprotective drugs [114]. Moreover, Wang et al. designed a PLT membrane coated with a PLT membrane suffixed with Arg-Gly-Asp (RGD) peptide and internally loaded with human fat extract (FE) PLGA particles (RGD-PLT@PLGA-FE) (Fig. 5C). FE contained various angiogenic factors, and RGD, an angiogenic peptide with active targeting ability, was able to deliver FE precisely. Ultimately, it provided therapeutic effects through revascularisation and neurogenesis in ischemic brain regions [115].

Ischemic and hypoxic changes in the intravascular microenvironment are often accompanied by activation of immune cells and upregulation of inflammatory factor expression [3, 9, 13]. Among them, neutrophils, as the earliest inflammatory response cells, have a strong tendency towards the site of ischemic injury. Based on this, Dong et al. investigated neutrophil-generated nanovesicles as a carrier capable of targeting ischemic stroke lesions [116]. The loaded Resolvin D2 (RvD2) was used to reduce immune cell-endothelial cell interactions and inflammatory cytokine production, thereby ameliorating vascular endothelial injury during ischemic stroke (Fig. 5D). In addition, Liu et al. took advantage of the unique biological properties of neutrophils to design a more complex “Nanobuffer” (Fig. 5E). The core of the Nanobuffer was a PLGA NP loaded with neuroprotective cannabidiol (CBD), and the surface was covered with a neutrophil membrane adorned with the antioxidant α-lipoic acid (LA), forming the basic unit of the biofilm (LA-NM-NP/CBD). The neighboring basic units were then triggered by ring-opening-polymerization (ROP), which induced the cross-fusion of the surface-coated neutrophil membranes to form the final “Nanobuffer” [117]. This drug delivery system built using neutrophil bionic technology not only has a protective effect on damaged neurons in the ischemic semidarktic band. It also enabled a long-lasting and slow release of CBD to improve the ischemic microenvironment and reduce infarct size. Similarly, monocyte membranes, which are similar to neutrophil membranes, can also be used as biofilm carriers for drug delivery. For example, Wang et al. designed a mononuclear cell membrane-encapsulated rapamycin nanoparticles (MCM/RNPs) drug delivery system (Fig. 5F). This system actively bounded to inflammatory endothelial cells, blocking monocyte-endothelial cell adhesion in the immune cascade response. Moreover, it could re-enter sites of endothelial injury to release rapamycin (RAP), inhibiting microglia proliferation and exerting anti-inflammatory and neuroprotective effects [118]. These innovative approaches held promise for the targeted treatment of ischemic stroke by leveraging the biology of immune cells and their interactions in the inflammatory response.

### Nucleic acid self-assembly nanocarrier delivery system

In recent years, many studies indicated that gene therapy was considered a promising treatment for ischemic stroke [119, 120]. Currently, viral vectors and non-viral vectors are mainly used to deliver genes to improve ischemic stroke. Compared to viral vectors, non-viral vectors, such as Poly-amidoamine dendrimer, have lower immunogenicity and toxicity [121]. However, their transfection efficiency is low. Therefore, many studies devoted to improving the gene transfection efficiency of non-viral vectors.

**Fig. 6:**
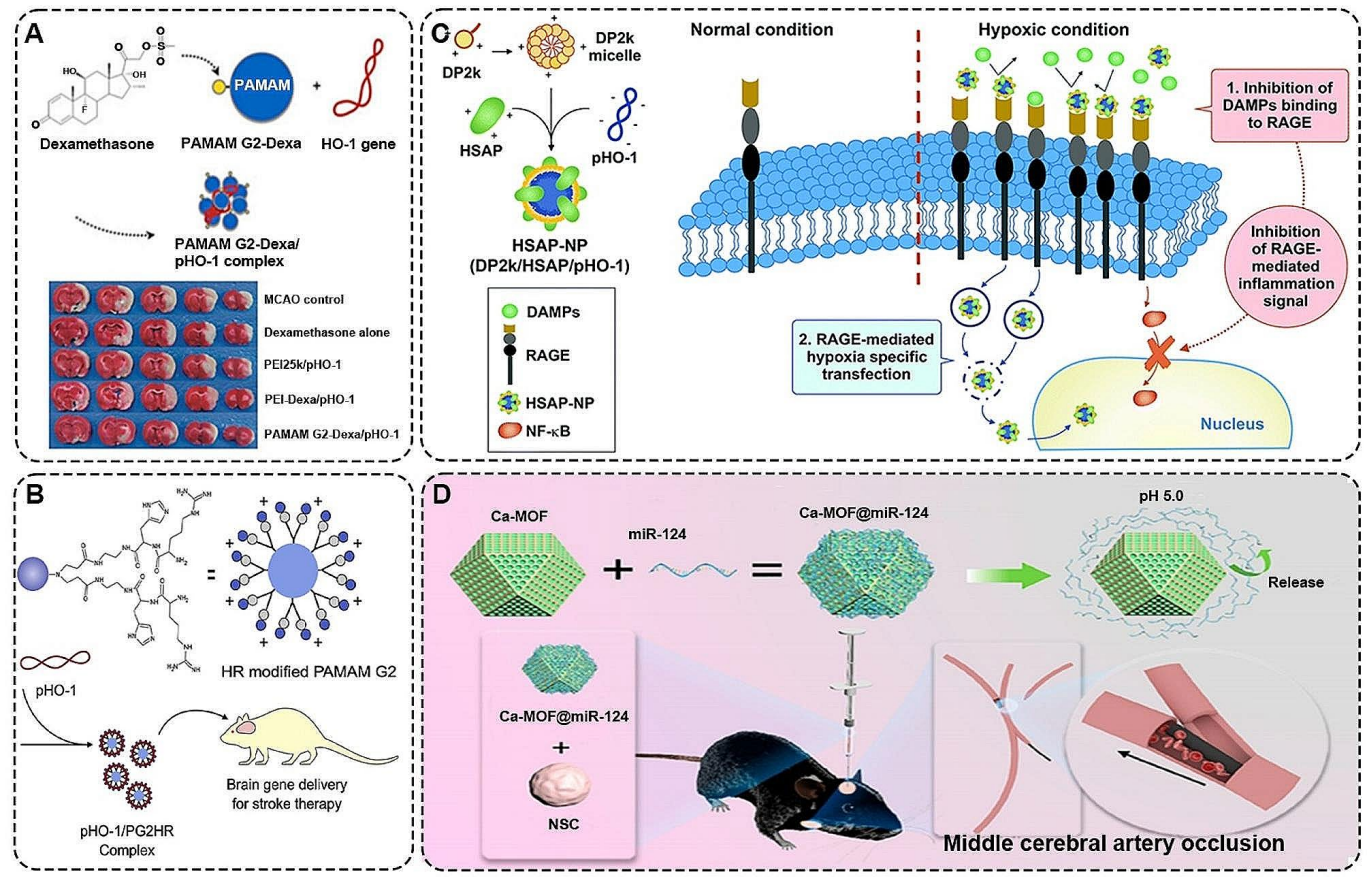
**A** Schematic illustration of the composition of the PAMAM G2-Dexa/pHO-1 complex and evaluation of the therapeutic efficacy of different constituent plasmid complexes in an animal model of stroke [123]. Copyright 2015 WILEY-VCH Verlag GmbH & Co. KGaA, Weinheim. **B** The pHO-1/PG2HR complex has low cytotoxicity and high gene delivery efficiency, making it more suitable for ischemic stroke inflammation-related gene therapy [124]. Copyright 2020, Elsevier B.V. **C** Schematic structure of self-assembled HSAP-NP [125]. Copyright 2019, The Royal Society of Chemistry. **D** Components and pH-responsive release characteristics of the Ca-MOF@miR-124 nano delivery system [127]. Copyright 2022, American Chemical Society

Poly-amidoamine dendrimer (PAMAM) is a dendrimer that is widely used in gene vectors to construct better anti-inflammatory constructs [122]. Based on this, Lee et al. used dexamethasone-conjugated poly-amidoamine generation 2 (PAMAM G2-Dexa) as a heme oxygenase-1 (HO-1) gene, which has anti-inflammatory and anti-apoptotic effects (Fig. 6A). In vivo results demonstrated that relative to Dexa alone, or the standard vector poly-ethylenimine (25 kDa) (PEI25k)/plasmid HO-1 (pHO-1), dexamethasone-conjugated PEI (PEI-Dexa)/pHO-1, this system was more efficiently transferred pHO-1 into the ischemic brain with higher therapeutic efficacy [123]. To further boost the transfection efficiency of the gene, the team also modified PAMAM G2 (PG2) by coupling histidine and arginine to the primary amine of PG2 and synthesized PG2HR (Fig. 6B). In vitro and in vivo results exhibited that PG2HR was a more efficient PHO-1 gene carrier, which could effectively treat ischemic stroke via reducing apoptosis and myocardial infarction area [124]. Notably, the team also constructed a bifunctional pHO gene vector formed by deoxycholate-conjugated polyethylenimine-2k (DP2k) and anti-RAGE peptide (HSAP) (Fig. 6C). On the one hand, the system inhibited RAGE-mediated inflammatory signaling by inhibiting damage-associated molecular patterns (DAMPs) from binding to RAGE. On the other hand, the vector enhanced gene transfection efficiency by increasing cellular uptake of nanoparticles into hypoxic cells via RAGE interactions in ischemic tissues [125]. Furthermore, the study suggested that mRNA had a lower inflammatory response, faster gene delivery and higher gene delivery efficiency relative to plasmid DNA. Based on this, the team constructed HO-1 mRNA/ PEI-Dexa [126]. Relative to pHO-1/PEI-Dexa, this complex showed a significant increase in transfection efficiency and expression rate and was effective in reducing the volume of cerebral infarction after cerebral ischemia. In a recent study, Yang et al. developed a Ca-Metal-organic frameworks (MOFs) (Ca-MOFs) @miR-124 nano delivery system to deliver miR-124 (Fig. 6D) [127]. This system effectively protected miR-124 from degradation by nucleases and promoted its internalization by neural stem cells. In vitro experiments demonstrated that the nanoparticles facilitated the differentiation of neural stem cells into mature neurons with electrophysiological functions within a short period. In vivo experiments showed that the combination of neural stem cells and Ca-MOF@miR-124 nanoparticles effectively reduced ischemic regions in the context of ischemic stroke.

### Atypical polymer nanocarrier delivery system

The investigation revealed that other structured nanocarriers have also been shown to be useful as drug carriers for use in the treatment of stroke, such as nanogels, nano emulsions, nanosheets (NSs) and carbonized polymer dots (CPDs).

**Fig. 7:**
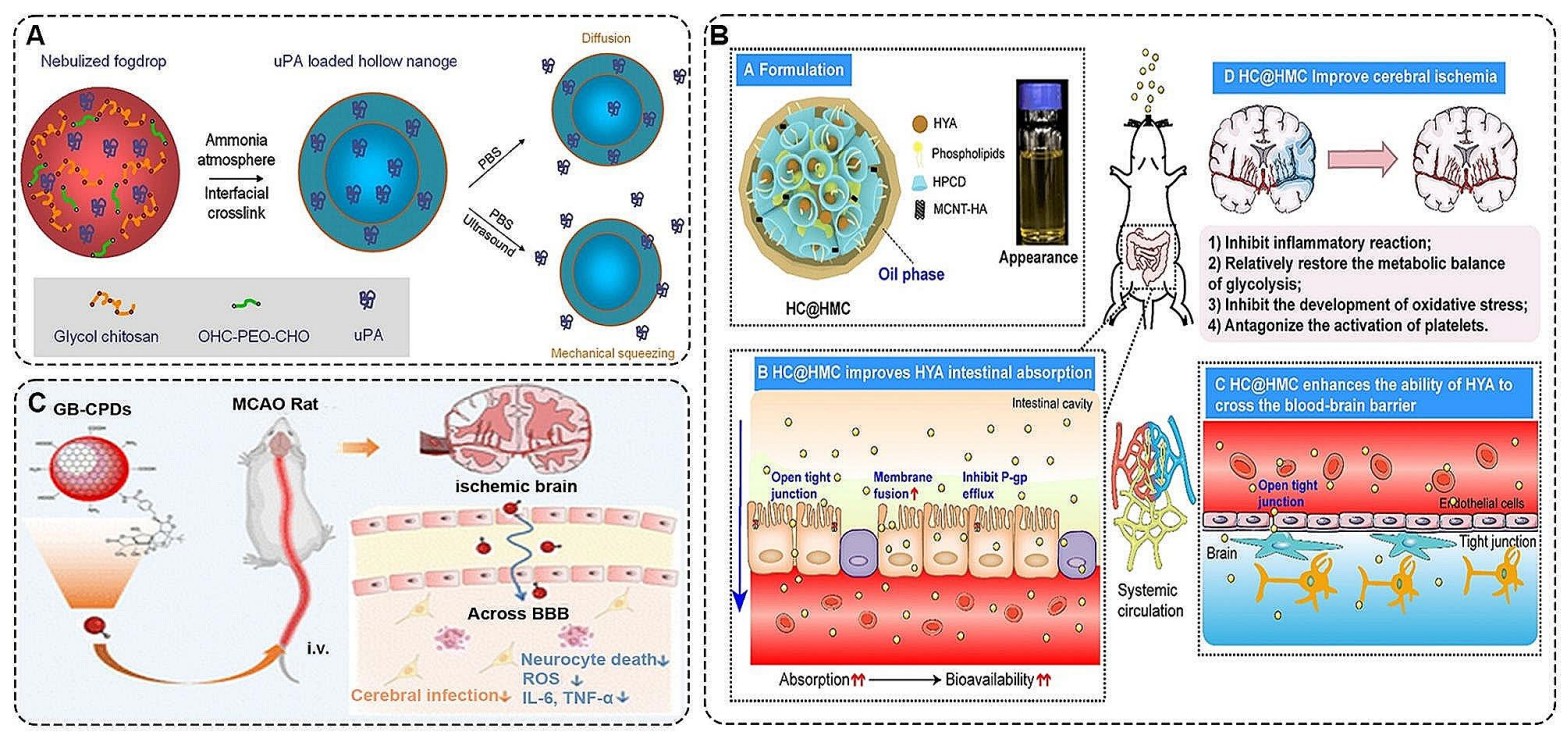
**A** Schematic representation of the synthesis of ultrasound responsive nanogel loaded with uPA [130]. Copyright 2012, Elsevier B.V. **B** Structural schematic and biological solubility of HC@HMC [134]. The intestinal absorption and pharmacokinetics of HC@HMC in rats were determined, and the specific protective mechanism of this nano emulsion against cerebral ischemia was also explored. Copyright 2023, MDPI. **C** GB-CPDs were able to cross the BBB and exert neuroprotective, ROS-scavenging and anti-inflammatory effects, ultimately reducing the volume of cerebral ischemic infarction [140]. Copyright 2023, The Royal Society of Chemistry

Nanogels, generally referred to as hydrogels with diameters below 200 nm, are characterized by higher specific surface area, volume effect and quantum size effect [128, 129]. Based on this, Jin et al. fabricated PEG-crosslinked glycol chitosan (GC) hollow nanogels loaded with thrombolytic-type fibrinogen activator (uPA) (Fig. 7A). In vitro and in vivo studies demonstrated that the lifespan of uPA was significantly prolonged in a rat model under the protection of a nanogel matrix and that ultrasound intervention effectively accelerates thrombolysis [130]. In addition, the team also established a combined system of pH response PEG-urokinase (UK) and free UK. This dual-targeted system both improved thrombolytic efficiency and offered the possibility of treating ischemic stroke beyond the time window [131]. Further mechanistic studies by the team indicated that PEG-UK was able to inhibit the activation of the LRP/NF-kB/COX-2 pathway, caspase cascade and NMDAR. Administration outside the normal time window still protected middle cerebral artery occlusion (MCAO) rats by maintaining the integrity of the BBB and inhibiting neurotoxicity and apoptosis [132].

Nano emulsions are two-phase dispersions of two incompatible liquids, consisting of nanoscale droplets with the advantages of slow release, accurate targeting, and lower biotoxicity. In addition, they increased the contact area of the drug with the gastric mucosa and enhanced the absorption and permeability of the BBB [133]. Considering these advantages, Zhang et al. prepared a nano-emulsion composed of hydroxysafflor yellow A (HYA) with phospholipid and HPCD complex and hyaluronic acid-modified multi-walled carbon nanotubes and chitosan (HC@HMC) by titration (Fig. 7B). The results showed that HC@HMC was able to ameliorate ischemic stroke by inhibiting the inflammatory response, restoring the metabolic balance of glycolysis, inhibiting oxidative response and activating platelets. It also improved the intestinal absorption of HYA and its ability to cross the BBB [134].

NSs are materials characterized by their single or multilayer sheet-like structures at the nanoscale, typically ranging in thickness from a few nanometres to tens of nanometres [135]. They possess remarkable modulability and targeting capabilities. Among these, black phosphorus nanosheets (BPNs) stand out as a prominent two-dimensional nano-delivery system with notable photothermal properties [136, 137]. They demonstrate the ability to traverse the blood-brain barrier under laser irradiation, offering potential for treating ischemic stroke. Wang et al. devised a novel neuroprotective nanomedicine by incorporating uPA onto BPNs. The experimental findings indicated that uPA-loaded BPNs not only efficiently delivered uPA for thrombus dissolution but also demonstrated the ability to scavenge ROS, thereby offering neuroprotection following uPA release [138].

CPD_S_ are an emerging class of carbon nanomaterials with dimensions less than 10 nm [139]. It has electrostatic interactions and BBB penetration capabilities that enable it to be used in the treatment of ischemic stroke. Specifically, Yang et al. constructed platelet-activating factor antagonist ginkgolide B (GB)-modified CPDs (GB-CPDs) and administered it cyclically to the MCAO rat model (Fig. 7C). The results revealed that GB-CPDS could cross the BBB to reach the brain and stay there for up to 72 h. Also, GB-CPDS remarkably reduced the area of cerebral infarcts in the MCAO rat model via antioxidant and anti-inflammatory [140].

## Drug release modalities from polymeric carriers

Drug release pattern is an important criterion for assessing the capability of drug delivery systems and is closely related to the efficacy of the drug exerted in the body after delivery [141–143]. Polymer nanocarriers are advanced drug delivery systems, ingeniously designed to amplify bioavailability, fortify stability, and hone the targeted delivery of therapeutic agents [144, 145]. These carriers are endowed with distinct, multifaceted release mechanisms, classified principally into passive drug release [146], stimulus-responsive drug release [147, 148], and targeted drug release [149]. Passive drug release encompasses conventional mechanisms such as diffusion and permeation, contingent upon concentration gradients and the inherent permeability of the polymeric matrix. The stimulus-responsive release, on the other hand, is meticulously orchestrated to coincide with specific physiological cues, leveraging pH variations, temperature fluctuations, redox changes, or magnetic fields to synchronize drug liberation with the pathophysiological context [150–152]. For instance, in the scenario of ischemic stroke, the overproduction of ROS is adeptly utilized to craft release systems that respond to oxidative cues [153]. Of paramount significance is the targeted drug release, which can be categorized into passive targeting, exploiting the enhanced permeability and retention (EPR) effect prevalent in pathological vasculature, and active targeting, which harnesses the specificity of ligand-receptor interactions to precipitate receptor-mediated endocytosis. The latter is achieved by adorning the surface of polymeric nanocarriers with ligands that exhibit high specificity towards receptors on targeted cells, thereby enhancing the selectivity and efficacy of the therapeutic delivery. In the polymer nano delivery system constructed for the treatment of ischemic stroke, there are drug release achieved through multiple modes, as described in Table 2.

**Table 2:** Drug release modalities from polymeric carriers

Type	Nanocarrier	Drug release mode	Refs.
Polymer Nanoparticles	RGD-DMAPA-Amyp	Neurovascular Targeted Release	[83]
	rtPA-associated Fuco-SPs		[91]
	A-BAM		[93]
	ASNPs		[71]
	E-A/P-CeO2		[73]
	Ac-PGP		[74]
	LEX@PLGA		[77]
	CAT&SOD@PLGA		[78]
	18β-GA-conjugated DEAE-DGA	ROS-Responsive Release	[87]
	AMD3100-conjugated PEG-PTT-T-PEG		[72]
	B-PDEA		[97]
	SAG@PHSRN-HES	pH-Responsive Release	[84]
	Aminated Chitosan		[85]
	rtPA-embedded PMNP	Magnetic Field-Responsive Release	[80]
	rmDPPs		[81]
Polymeric Micelles	EDV-AM	Neurovascular Targeted Release	[99]
	ISL-M		[103]
	CREKA-PEG-LysB	Neurovascular Targeted and ROS-Responsive Release	[105]
	SHR	pH-Responsive Release	[101]
	Res-loaded cRGD/TPP		[102]
Biolipid-coated Polymer Nanocarriers	PNP-PA	Neurovascular Targeted Release	[113]
	tP-NP-rtPA/ZL006e		[114]
	RGD-PLT@PLGA-FE		[115]
	RvD2-loaded Nanovesicles		[116]
	LA-NM-NP/CBD		[117]
	MCM/RNPs		[118]
	SHp-RBA-NP	ROS-Responsive Release	[112]
Nucleic Acid Self-assembling Nanocarriers	PEI-Dexa/pHO-1	Neurovascular Targeted Release	[123]
	DP2k/HSAP/pHO-1		[125]
	HO-1 mRNA/ PEI-Dexa		[126]
Atypical Polymer Nanocarrier Systems	HC@HMC		[134]
	GB-CPDs		[140]
	PEG-UK	pH-Responsive Release	[132]

## Conclusions and perspectives

Ischemic stroke is a severe cerebrovascular disease that arises from diverse pathogenic mechanisms, including but not limited to neuronal damage, imbalances in acid-base metabolism, alterations in ion metabolism, and activation of inflammatory cascade responses. These intricate pathological processes, along with the densely packed structure of the blood‒brain barrier and the inadequacy of conventional treatment methods, frequently lead to unfavorable treatment outcomes and prognoses.

However, in recent years, nanotechnology has garnered significant attention in the medical domain, particularly in the context of stroke and cerebrovascular disorders, providing a promising avenue for addressing these challenges. Nanomaterials possess considerable versatility, and their surface properties can be optimized through surface biomodification, which is essential for targeted drug delivery. They exhibit high encapsulation efficiency, large loading capacity, and controlled release properties, enabling regulation of drug concentration and accumulation at the site of the lesion. Moreover, nanomaterials can improve pharmacokinetics by extending the half-life of drugs in circulation and enhancing permeability due to their innate biocompatibility. Among various nanomaterials, polymeric nanoparticles are extensively utilized for stroke treatment, as they produce more stable drug delivery systems that are readily accessible. Drug loading onto polymer nanocarrier delivery system can be accomplished through covalent bonding, adsorption, or encapsulation. An all-encompassing drug delivery system enables preferential release at the target site, controlled drug release over an extended duration, maintenance of drug concentration within the appropriate range of the focal tissue, and protection of the drug from enzymatic degradation and rapid clearance in vivo.

Polymeric nanocarriers, as a maturing drug delivery system, have been prepared and constructed in a variety of ways. We broadly categorize the preparation methods of polymeric nanodrug delivery systems mentioned above in the field of ischemic stroke therapy, which mainly include nanoprecipitation, solvent exchange, emulsion polymerization, ionotropic gelation, and self-assembly mechanisms. However, these conventional methods often fall short in enabling the scale-up necessary for widespread clinical application and mass production. Notable limitations include reliance on hazardous solvents, rigorous purification requirements, and the challenges associated with uniform particle functionalization at an industrial scale. Consequently, there is a pressing need to embrace and refine emerging technologies and enhance process innovation, such as leveraging supercritical fluid technology for solvent-free synthesis [154, 155] and employing electrospinning [156] for the mass production of nanofibers. Future prospects include harnessing 3D printing for the precise fabrication of polymeric nanocarriers and exploring biomimetic approaches to foster more sustainable and universally applicable mass production techniques.

Despite the growing application of nanomaterials for the management of brain diseases, the mechanisms governing drug transport through the BBB remain largely unexplored. While molecular modification and drug release kinetics may influence the overall drug delivery system, the biotoxicity and cytocompatibility of the nanomaterials and their functionalization should not be overlooked. To promote and advance the application of nanomaterials in the treatment of brain diseases, it is imperative to integrate and coordinate the drug delivery route, the molecular composition of nanocarriers, and the drug delivery time. In the context of stroke treatment, determining the pathological state of the disease is crucial to regulating the drug within the effective therapeutic time window. With regard to the composition of the drug delivery system, the amount of drug that can be absorbed by the system and the timing of release remains uncertain. Addressing these research gaps and striving for a more comprehensive understanding of the system will significantly enhance its application in the treatment of ischemic stroke.

## Data Availability

No datasets were generated or analysed during the current study.

## Abbreviations

BBB: Blood-brain barrier
ROS: Reactive oxygen species
rtPA: Recombinant tissue plasminogen activator
ICAM-1: Intercellular cell adhesion molecule-1
LFA-1: Lymphocyte function-associated antigen-1
Mac-1: Macrophage-1
NMDA: N-methyl-D-aspartate
AMPA: Alpha-amino-3-hydroxy-5-methylisoxazole-4-propionic acid
PEG: Polyethylene glycol; PLA: polylactic acid; PLGA: polylactic-co-glycolic acid
HIF-1α: Hypoxia-inducible factor-1α
HES: Hydroxyethylstarch
SAG: Smoothened agonist
PHSRN: Pro-His-Ser-Arg-Asn
GA: Glycyrrhetinic acid
DEAE: Diethylaminoethylen
DGA: Dextran nanoparticles
BA: Betulinic acid
BAM: Acidic microenvironment of botulin
A-BAM: AMD3100 conjugated betulinicamine
PCL: Poly(ε-caprolactone)
PTT: PEG-poly(2-methylene-thiodiethylene-3-thiodipropionate)
E-A/P-CeO_2_: Edaravone-carried and PEG/Angiopep-2(ANG)-conjugated ceria nanoparticles
Ac-PGP: Acetyl Pro-Gly-Pro
DGL: Dendritic graft poly L-lysine
CAT: Catalase
H-MnO_2_: Hollow-structured MnO_2_
CBSA-PEG-TIIA-NPs: Cationic bovine serum albumin-conjugated tanshinone IIA PEG-Conjugated nanoparticles
CTX: Chloramphenicol
LEX: Lexiscan
SOD: Superoxide dismutase
PNAs: Peptide nucleic acid
PS: Phosphorothioates
MNP: Magnetic nanoparticles
PMNP: PLGA MNP
SPIONs: Superparamagnetic iron oxide nanoparticles
rmDPPs: rtPA and superparamagnetic iron oxide nanoparticles
PDMS: Semi-permanent polydimethylsiloxane
PVA: Polyvinyl alcohol; NSCs: neural stem cells
B-PDEA: Charge-reversal poly[(2-acryloyl) ethyl (p-boronic acid benzyl) diethylammonium bromide
pBDNF: BDNF plasmid
EDV: Edaravone
EDV-AM: Edaravone-agonistic micelle
SHR: SS-31-hyaluronicacid-rutin polymeric micelle
cRGD: Cyclo(Arg-Gly-Asp-DTyr-Lys) peptide
TPP: Triphenylphosphine
Res: Resveratrol
ISL-M: Angiopep-2-modified DSPE-PEG200
ISL: Isoliquiritigenin
mTOR: Mammalian target of rapamycin
tPA-DPNs: tPA with the porous structure of soft discoidal polymeric nanoconstructs
SHp: Stroke homing peptide
SHp-RBA-NP: A boric acid-modified dextran polymer core and a shell made of erythrocyte membrane surface-affixed with stroke homing peptide
NMDAR: N-methyl-D-aspartate receptor
PSD-95: Postsynaptic density protein
m-dextran: acetal-modified dextran
RGD-PLT@PLGA-FE: A PLT membrane suffixed with Arg-Gly-Asp (RGD) peptide and internally loaded with human fat extract (FE) PLGA particles
RvD2: Resolvin D2
CBD: Cannabidiol
LA: α-lipoic acid
ROP: Ring-opening-polymerization
MCM/RNPs: A mononuclear cell membrane-encapsulated rapamycin nanoparticles
RAP: Rapamycin
PAMAM: Poly-amidoamine dendrimer
PAMAM G2-Dexa: Dexamethasone-conjugated poly-amidoamine generation 2
HO-1: A heme oxygenase-1
PEI25k: Poly-ethylenimine (25 kDa)
pHO-1: Plasmid HO-1
PEI-Dexa: Dexamethasone-conjugated PEI
PG2: PAMAM G2
DP2k: Deoxycholate-conjugated polyethylenimine-2k
HSAP: pHO gene vector formed by DP2k and anti-RAGE peptide
DAMPs: Damage-associated molecular patterns
MOFs: Metal-organic frameworks
Ca-MOFs: Ca-Metal-organic frameworks
CPDs: Carbonized polymer dots
GC: Glycol chitosan
UK: Urokinase
MCAO: Middle cerebral artery occlusion
HYA: Hydroxysafflor yellow A
HC@HMC: Hyaluronic acid-modified multi-walled carbon nanotubes and chitosan
GB-CPDs: Ginkgolide B (GB)-modified CPDs
